# Psychometric Properties of the 20-Item Toronto Alexithymia Scale in the Chilean Population

**DOI:** 10.3389/fpsyg.2018.00963

**Published:** 2018-06-12

**Authors:** Mauricio González-Arias, Agustín Martínez-Molina, Susan Galdames, Alfonso Urzúa

**Affiliations:** ^1^Psicología, University of La Serena, La Serena, Chile; ^2^Metodología de las Ciencias del Comportamiento, Psicología y Sociología, Universidad de Zaragoza, Zaragoza, Spain; ^3^Psicología, Universidad Católica del Norte, Antofagasta, Chile

**Keywords:** alexithymia, emotion, TAS-20, psychometric properties, ESEM, wording factor

## Abstract

Alexithymia can be defined as inability to identify and describe emotions in the self. Has shown to be related to several psychological and pathological processes that can result in unsatisfactory interpersonal relationships and decreased social adjustment. Advances in research of alexithymia require the development and validation of assessment instruments, and its application to different population. With this aim, we studied the psychometric properties of the Twenty-Item Toronto Alexithymia Scale (TAS-20) in Chilean population using various modeling procedures (e.g., CFA, ESEM) in different structures (i.e., Correlated, Unidimensional, Hierarchical or Wording factors). Among the 10 models tested, the four-dimensional structure offered the best fit but with item-loading problems in the last factor (Pragmatic Thinking). We suggest that the studied version of the scale needs improvement (theoretical and empirical) to ensure optimal indices of validation for Chilean population.

## Introduction

The concept of Alexithymia (Sifneos, 1973) can be defined as cognitive and an affective deficit in the way that some individuals recognize and communicate emotional states (Taylor, 1984). This concept has proved to be related to several psychological and pathological processes such as: interoceptive awareness (Herbert et al., 2011), traumatic memories (Nandrino et al., 2006), suicide risk (De Berardis et al., 2017), depression (Arancibia and Behar, 2015; Melin et al., 2017), eating disorders (Behar et al., 2014), somatoform and conversion disorders (Arancibia et al., 2016), chronic pain (Saariaho et al., 2017) and also psychosomatic illness (Kano and Fukudo, 2013; Porcelli et al., 2017), among others. Resulting in unsatisfactory interpersonal relationships and decreased social adjustment (Taylor and Bagby, 2012). Alexithymia has also been associated with different kinds of addictive disorders, such as pathological gambling (Lumley and Roby, 1995; Maniaci et al., 2017), Internet addiction (Dalbudak et al., 2013; Scimeca et al., 2014), maladaptive sexual behavior (Scimeca et al., 2013), and abnormal illness behavior (Scimeca et al., 2016).

Taylor (1984), basing on a literature review of alexithymia, proposes a scale to measure this construct (Toronto Alexithymia Scale-TAS-26), based on five dimensions: (1) difficulty identifying feelings and distinguishing between feelings and bodily sensations of emotional arousal; (2) difficulty describing feelings to others; (3) externally oriented thinking (or lack of introspection); (4) social conformism and (5) lack of daydreaming and other imaginative activity (Taylor et al., 1985).

Later, Bagby et al. (1994) proposes a revised brief version of this self-report Likert scale: the TAS-20. This scale conserved only the first three traits as factors. In some way, the last two traits remained present in factors 2 and 3 as a more general operatory thinking component oriented to the preference for the external details of everyday life instead of thought content related to inner experience.

TAS-20 has been validated in clinical and non-clinical population, including mental and chronic physical illness. As point out by Taylor et al. (2003), the English version of TAS-20 has been translated to many different languages. In the last years, there have been validation reports in Arabic population (El Abiddine et al., 2017), Chinese population (Zhu et al., 2007), Croatian (Kocijan et al., 2015), Portuguese (Brazil) (Yoshida, 2007), Greek (Tsaousis et al., 2010), Dutch (Adolescents) (Meganck et al., 2012), and Turkish population (Gülec et al., 2009; Bolat et al., 2017). In Latin-American countries there has been reported a Peruvian (Loiselle and Cossette, 2001) and two Mexican versions (Pérez-Rincón et al., 1997; Moral, 2010).

In the psychometric field, the TAS-20 has demonstrated good internal consistency and test–retest reliability. The validation studies yielded to a three-factor structure congruent with the theoretical construct of alexithymia and this structure remains relatively stable in several cultures and languages (Taylor et al., 2003). In addition, it has been shown invariance of the three factors between men and women (Parker et al., 2003). Despite, is also possible to find research reports with results that show good fit indicators for four or more factors models (Tsaousis et al., 2010; Meganck et al., 2012).

Evidence has been found about the possible role of culture in the factorial structure. Culhane et al. (2009), presented evidence of invariance comparing US-Anglo and US-Hispanic student samples. On the opposite, Peruvian (Loiselle and Cossette, 2001) and Mexican (Pérez-Rincón et al., 1997; Moral, 2010) studies showed poorer fit indices. These different findings open the question about the possible role of culture in the factorial structure. The Peruvian sample contrasted a three-factor model using Confirmatory Factor Analysis. These authors reported lack of fit, particularly in the third factor, and they mention as possible reasons that this problematic factor includes 4 negative keyed items (4 of the 5 total, and 4 of 8 of the third factor), which they think it could mean a greater difficulty in answering these items and a low reliance on introspection when describing affective states. With this respect, Fernández-Jiménez et al. (2013) says that these Spanish adaptations of the scale have certain limitations: (a) the Mexican and Peruvian versions present some local particularities in language use, when compared with the Spanish spoken in Spain; (b) Latin-American versions, and the version developed in Spain, contain some items whose back-translation does not adequately reflect the meanings of the original English version of the items; (c) the indices to assess the fit of the proposed models do not meet the standards that are currently recommended (CFI ≥ 0.95, TLI ≥ 0.95, and RMSEA < 0.06; Hu and Bentler, 1999; Schreiber, 2017). Moreover, the size of the sample with which the psychometric properties of Spanish version were supported was tight for some of the tested models according to Wolf et al. (2013) indications.

In Chile, only one undergraduate thesis was found to evaluate the reliability and validity of the TAS-20 in 236 university students in the city of Chillán (Sáez and Tiznado, 2012). However, only a principal component analysis was applied in this study.

Advances in research of alexithymia require the development and validation of assessment instruments, and its application to different population. With this aim, we studied the psychometric properties of the Twenty-Item Toronto Alexithymia Scale (TAS-20), which now is, the most widely used instrument to measure Alexithymia. We have applied this scale to Chilean university students and we performed analysis using different model testing procedures.

## Materials and Methods

### Participants

A total of 516 students voluntarily participated in this study. Most were female (*n* = 340, 65.8%, *Mean Age* = 22; *SD* = 5.1), and 176 were males (34.2%, *Mean Age* = 22; *SD* = 3.7). 54.7% (*n* = 282) of the students were from the cities of La Serena and Coquimbo, 8.9% (*n* = 46) from Iquique, 9.1% (*n* = 47) from Antofagasta, 8.1% (*n* = 42) from Santiago, 9.5% (*n* = 49) from Temuco and 9.7% (*n* = 50) from Punta Arenas, throughout Chile. They all spoke Spanish as their mother tongue.

### Measures

The English version of the TAS 20 (see **Table 1**; Meganck et al., 2008) was translated and adapted to the Spanish language following the international guidelines (Hambleton, 1994; International Test Commission, 2010).

**Table 1:** Items of the TAS-20 Spanish version.

Number	Dificulty identifying feelings (Dificultad identificando sentimientos)
1	A menudo estoy confuso con las emociones que estoy sintiendo
3	Tengo sensaciones físicas que ni incluso los doctores entienden
6	Cuando estoy mal no se si estoy triste, asustado o enfadado
7	A menudo estoy confundido con las sensaciones de mi cuerpo
9	Tengo sentimientos que casi no puedo identificar
13	No sé qué pasa dentro de mi
14	A menudo no se por qué estoy enfadado
**Number**	**Dificulty describing feelings (Dificultad describiendo sentimientos)**
2	Me es difícil encontrar las palabras correctas para mis sentimientos
*4*	*Soy capaz de expresar mis sentimientos fácilmente*
11	Me es difícil expresar lo que siento acerca de las personas
12	La gente me dice que exprese más mis sentimientos
17	Me es difícil revelar mis sentimientos más profundos incluso a mis amigos más cercanos
**Number**	**Externally oriented thinking (Pensamiento orientado al exterior)**
*5*	*Prefiero analizar los problemas mejor que sólo describirlos*
8	Prefiero dejar que las cosas sucedan solas, mejor que preguntarme por qué suceden de ese modo
*10*	*Estar en contacto con las emociones es esencial*
15	Prefiero hablar con la gente de sus actividades diarias mejor que de sus sentimientos
16	Prefiero ver espectáculos simples, pero entretenidos, que dramas psicológicos
*18*	*Puedo sentirme cercano a alguien, incluso en momentos de silencio*
*19*	*Es útil examinar mis sentimientos para resolver problemas personales*
20	Buscar significados ocultos a películas o juegos disminuye el placer de disfrutarlos

*Translated and adapted from Meganck et al. (2008); *italic items* = negatively keyed.*

This Spanish version of TAS-20 includes 20 self-report questions distributed into three subscales: (1) difficulty identifying feelings and distinguishing between feelings and bodily sensations in emotional activation, (2) difficulty in the verbal expression of emotions, and (3) externally oriented thinking. The answers values fluctuate between 1 and 5 points (1 is the lack of it and 5 is most present), and items 4, 5, 10, 18, and 19 must be inverted before adding up scores. Total score interval is 20–100, while a person is considered alexithymic with a score ≥ 61.

### Procedure

The students voluntarily completed the scales after reading and written informed consents. All procedures in this study followed (a) the principles of Helsinki Declaration (World Medical Association, 2013), (b) the APA ethical standards (Including 2010 and 2016 Amendments), and (c) the guidelines of the National Commission for Scientific and Technological Research of Chile (CONICYT). There were no missing data in this study.

### Tested Models

The six basic models tested by Meganck et al. (2008, 2012) were compared using Confirmatory Factor analysis (see **Table 2**). The first Model (a) is proposed as a unidimensional structure where all items reflect alexithymia. Model (b) is a two-factor structure with DIF and DDF items forming one factor and EOT items forming the second factor (Haviland and Reise, 1996; Loas et al., 1996; Erni et al., 1997). Model (c) (Kooiman et al., 2002) proposed the same structure of model (b) but with only 16 items (items 16, 17, 18, and 20 were erased). The fourth model (d) is composed by three factors: DIF, DDF, and EOT (Bagby et al., 1994) and the fifth model (e) is a three-factor solution (Ritz and Kannapin, 2000); DIF and DDF items as one factor and EOT split into two factors (PR and IM). Finally, the sixth model (f) is a four-factor solution that considers the dimensions DIF and DDF plus de sub-dimensions PR and IM that were split from EOT (Müller et al., 2003).

**Table 2:** Basic models of the TAS-20 (proposed in previous literature).

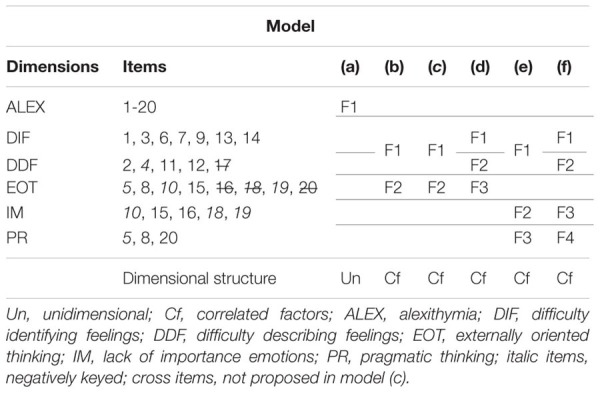

Further models were also tested in this study for those models described above that provided adequate fit to data: hierarchical (Hi), wording factor (Wf), and exploratory structural exploratory models (ESEMs). When the relationship between first-order factors is high, hierarchical models of indirect effects could be proposed. The Hi second-order structure was tested with the general alexithymia concept as a higher level.

The choice of one model or another is a theoretical, applied, and parsimony-based decision. The Wf was tested as an orthogonal method factor (bifactor) on which the negatively keyed items are located (4, 5, 10, 18, and 19). Finally, ESEM approach integrates the flexibility of EFA and the advantages of CFA (Asparouhov and Muthén, 2009; Garrido et al., in press). Even if these models are not contemplated in the classical frameworks (models 1–6), the exploratory approach could end up suggesting a more efficient latent structure than those that have been derived from previous studies (e.g., less or specific dimensions in a bifactor structure; Arias et al., 2016).

### Data Analysis

We firstly explored the reliability and adequacy of factor analysis indices for each TAS-20 scale. These statistics were: the explained proportion of variance (PEV), Barlett’s test and KMO index for the adequacy of the analysis, the number of advised dimensions in each scale with the parallel analysis technique (Garrido et al., 2013), Cronbach’s α and McDonald’s ω as an estimate of the reliability. For this purpose, we employed an unweighted least-squares (ULS) estimator based on polichoric correlations because of the ordinal nature of data. The use of robust estimators as ULS are recommended because they may produce more accurate parameter estimates than Maximum Likelihood (Forero et al., 2009; Schmitt, 2011; Li, 2014). If necessary an oblique rotation was used because of possible secondary and related dimensions (Promin; salient larger values > 0.30; Lorenzo-Seva, 1999).

In addition to the descriptive and complementary factorial indices of the scales, different latent structures were tested (CFA and ESEM). These models were executed using the same factorial considerations as the previous (polichoric correlations and ULSMV estimator available in Mplus version 7.4). Cut-off point recommendations of Schreiber (2017) were followed for goodness of fit indices criteria: CFI ≥ 0.95, TLI ≥ 0.95, and RMSEA < 0.06.

## Results

**Table 3** shows descriptive statistics computed with SPSS 22. The average values of the scales showed a similar central tendency to those reported by previous studies for students (Taylor et al., 2003; Meganck et al., 2012). However, the variance of the items was slightly higher in the sample of Chilean students. The most closely related scales were DIF and DDF on the one hand, and IM and PR on the other.

**Table 3:** Descriptive statistics and Pearson correlations for the TAS-20 scales.

	ALEX	DIF	DDF	EOT	IM	PR
ALEX						
DIF	0.797*					
DDF	0.794*	0.585*				
EOT	0.684*	0.209*	0.301*			
IM	0.637*	0.171*	0.307*	0.934*		
PR	0.514*	0.201*	0.175*	0.747*	0.461*	

n ítems	20	7	5	8	5	3
*M*	53.11	16.37	15.25	21.49	13.66	7.83
*SD*	14.14	6.84	5.24	6.67	5.00	2.68
Skewness	0.27	0.77	0.29	0.32	0.34	0.30
Kurtosis	–0.23	0.17	–0.35	–0.64	–0.78	–0.39

*ALEX, alexithymia; DIF, difficulty identifying feelings; DDF, difficulty describing feelings; EOT, externally oriented thinking; IM, lack of importance emotions; PR, pragmatic thinking. ^∗^*p* < 0.01.*

DIF and DDF proposed scales (**Table 4**) had good exploratory values to be considered as possible single dimensions (one advised dimension per scale, adequate proportions of explained variance, Bartlett test *p* < 0.01 and KMO ≥ 0.8; Lorenzo-Seva and Ferrando, 2013). EOT, IM, and PR scales did not have adequate exploratory values to be considered as possible single dimensions. It should be noted its lack of reliability (α or ω < 0.70). Note that these factors reflect a small number of items (especially PR).

**Table 4:** Reliability and adequacy factor analysis indices for the TAS-20 proposed scales.

	ALEX	DIF	DDF	EOT	IM	PR
n items	20	7	5	8	5	3
AND	4	1	1	2	2	2
PEV	0.59	0.54	0.51	0.36	0.47	0.40
Bartlett (*df*)	3792.6 (190)^∗^	1362.1 (21)^∗^	487.3 (10)^∗^	1252.4 (28)^∗^	790.5 (10)^∗^	13.2 (3)^∗^
KMO	0.869	0.898	0.767	0.786	0.835	0.503
Cronbach’s α	0.829	0.855	0.730	0.663	0.650	0.102
McDonald’s ω	0.838	0.861	0.764	0.690	0.677	0.311

*ALEX, alexithymia; DIF, difficulty identifying feelings; DDF, difficulty describing feelings; EOT, externally oriented thinking; IM, lack of importance emotions; PR, pragmatic thinking; AND, advised number of dimensions by Horn’s parallel analysis; PEV, proportion of explained variance; Barlett, Barlett’s test; KMO, Kaiser-Meyer-Olkin Index. ^∗^*p* < 0.001.*

Reliability indices of the TAS-20 scales were similar to those reported by Bagby et al. (1994) and Meganck et al. (2012). Both DIF and DDF tend to show good magnitudes of reliability, whereas EOT (and its sub-factors IM and PR), as previous studies, showed inadequate reliability indices (<0.80).

All confirmatory modeling analyzes on the basic models reported poor or not adequate fit indices (**Table 5**). Only models (c) and (f) reached an acceptable CFI value (>0.90). Models (d) and (f) referred the lowest RMSEA. In relation to the other basic models and considering the set of indicators (χ^2^, RMSEA, CFI, and TLI), the best structure for these datasets was four correlated factors (f).

**Table 5:** Fit indices for the basic models of the TAS-20.

Model					Fit índices					
		s	Dimensions	i	χ^2^	*df*	χ^2^/df	CFI	TLI	RMSEA
1 (a)	CFA	Un	ALEX	20	1955,11	170	11.50	0.612	0.566	0.143
2 (b)	CFA	Cf	DIF/DDF-EOT	20	1000,04	169	5.92	0.819	0.797	0.098
3 (*c*)	CFA	Cf	DIF/DDF-EOT	16	786,10	103	7.63	0.908	0.893	0.113
4 (d)	CFA	Cf	DIF-DDF-EOT	20	918,69	167	5.50	0.837	0.814	0.093
5 (e)	CFA	Cf	DIF/DDF-PR-IM	20	997,93	167	5.98	0.819	0.794	0.098
6 (*f*)	CFA	Cf	DIF-DDF-PR-IM	20	904,80	164	5.52	0.938	0.813	0.094

*s, structure; Un, unidimensional; Cf, correlated factors; i, number of items; CFI, Comparative Fit Index; TLI, Tucker-Lewis Index; RMSEA, root-mean-square error of approximation.*

We also tested two bifactor models with the purpose of exploring other dimensional structures (correlated and uncorrelated specific factors together with a general factor of alexithymia). These tested solutions were uninterpretable, that is, poor fit indices (i.e., RMSEA > 0.10) and mostly low or negative factor loadings.

CFA analysis considering Hi or Wf structures in model (f) did not meet acceptable fit indices. As expected, the alternative ESEM approach of the TAS-20 was supported by good fit indices (χ^2^/df < 0.3, RMSEA < 0.06, CFI and TLI > 0.90). Although not shown in **Table 6**, the rest of the basic models (a–e) were also tested with an ESEM approach. None of these models reported better ESEM fit indices than the (f).

**Table 6:** Fit indices for the alternative structures of the TAS-20.

Model					Fit índices					
		s	Dimensions	i	χ^2^	*df*	χ^2^/df	CFI	TLI	RMSEA
7 (f)	CFA	Hi	DIF-DDF-PR-IM	20	1259,64	166	7,59	0.762	0.728	0.113
8 (f)	CFA	Wf	DIF-DDF-PR-IM	20	785,05	160	4,91	0.870	0.846	0.085
9 (f)	ESEM	Cf	DIF-DDF-IM-PR	20	273,65	116	2,36	0.966	0.944	0.051
10 (f)	ESEM	Wf	DIF-DDF-IM-PR	20	238,45	112	2,13	0.972	0.953	0.047

*s, structure; CFA, confirmatory factor analysis; ESEMs, exploratory structural equation models; Hi, hierarchical alexithymia 2^*nd*^ order factor; Wf, wording factor on which the negatively keyed items load (Items *4, 5, 10, 18*, and *19*); Cf, correlates factors; i, number of items; CFI, Comparative Fit Index; TLI, Tucker-Lewis Index; RMSEA, root-mean-square error of approximation.*

**Table 7** shows the estimates of model 10 (f) (with the best fit-indices among the tested). In the table we can see clearly how the estimates loaded mainly in their corresponding factors (≥0.30) except for the fourth factor. PR was defined mainly by one loading (20) and two cross-loadings (15 and 16). Item 8 did not load substantially on any factor of the extracted ones.

**Table 7:** ESEM estimates for (f) + Wording factor.

		Model 10 (f) + Wf			
D	i	F1	F2	F3	F4
	01	**0.65**	0.19	–0.08	–0.07
	03	**0.55**	–0.01	–0.01	0.04
	06	**0.76**	0.04	–0.03	0.03
DIF	07	**0.85**	–0.04	–0.06	0.05
	09	**0.84**	0.02	–0.02	0.02
	13	**0.65**	0.19	0.08	0.01
	14	**0.54**	0.17	0.09	0.03

	02	**0.48**	**0.32**	0.03	–0.08
	04^∗^	0.02	**0.62**	–0.29	0.00
DDF	11	0.24	**0.51**	0.12	–0.07
	12	0.01	**0.66**	0.08	–0.09
	17	–0.03	**0.63**	0.29	0.08

	10^∗^	–0.03	–0.02	**0.60**	–0.15
	15	–0.11	0.35	**0.39**	**0.38**
IM	16	0.03	–0.01	**0.31**	**0.59**
	18^∗^	0.03	–0.01	**0.48**	–0.25
	19^∗^	–0.01	0.03	**0.64**	–0.24

	05^∗^	0.20	–0.23	**0.43**	0.26
PR	08	0.20	0.00	0.17	–0.26
	20	0.19	–0.16	0.07	**0.44**

	F2	0.53			
	F3	0.01	0.17		
	F4	0.27	0.10	–0.05	

*D, dimension; i, item number; *^∗^*negatively keyed items. Loadings greater than 0.30 are in bold. Correlations between factors are presented in the lower part of the table.*

## Discussion

A Chilean version of TAS-20 has been studied and the results show evidence of its reliability and construct validity, detecting some problems that must be addressed in future studies (e.g., items 5, 8, and 15 could be complicated to translate into Spanish because it requires the interpretation of “mejor que” [best than] in the sense of “en vez de” [instead of]; or item 10 in our culture the meaning of “estar en contacto” could be interpreted as a more concrete physical touch and therefore its comprehension requires the capacity to understand a metaphor, which is not of a common sense use).

Among the 10 tested models, including a unidimensional factor or the combination of different factors, we only found relatively good fit estimates for a model of four factors. These results do not follow the direction of some previous studies in English speaking population (that support a three-factor structure). They rather show consistency with the results observed in Latino-American population reported by Loiselle and Cossette (2001) in a Peruvian sample and Moral (2010) in a Mexican sample. Compared to these studies there is also a difference to be considered, that is, the fourth factor shows weaker indices of fit in our study. As a possible explanation of differences, we adhere to Taylor et al. (2003) hypothesis that points out that differences between Latino-American and Anglo-American population could be due to translation problems or related to cultural aspects of alexithymia in Latino-American population. The third factor structure not only has been problematic in Latinamerica. Moreover, this can be enhanced by (a) respondent’s low reading comprehension skills, particularly affecting negatively keyed items and those that include more abstract ideas, (b) a culture that is not used to make verbal language distinctions between “internal and external” world, and (c) the presence of patriarchal cultural beliefs that contradict the idea of affectivity as a positive domain. These beliefs usually see emotions as dangerous and to be controlled (Arón, 2001; Blanco and De la Corte, 2003).

On the other hand, it could be as in previous studies, that the psychometric properties of some factors were not adequate (Zhu et al., 2007). For this reason, it is understandable that structures modeled with CFA forcing IM and PR dimensions regularly show inadequate adjustment. As an alternative analysis, (f) ESEM model showed the best-fit indices in comparison with previous studies. In this sense the work of Craparo et al. (2015) already showed an oblique solution that substantially improves the fit of a TAS-20 structure.

The improvements that we recommend include the revision of dimensions IM and PR items, particularly item number 8, for its lack of load on the extracted factors. It may be necessary to reduce the scale. Gori et al. (2012) measured alexithymia and reported good reliability and validity indices with only five items (the PTI-Alexithymia Scale; “PTI-AS”). The PTI-AS highly correlates with TAS-20.

A limitation of this study is that the sample includes only university students, so it is not generalizable to the entire population and it is not directly comparable with studies performed with samples of young adolescents or clinical population. Given the use of a non-probabilistic and homogeneous sample for this study, more empirical support of the proposed factorial solution of this study with Chilean samples is necessary (e.g., young adolescents or clinical population).

## Conclusion

We found evidence that reliability and construct validity of TAS-20 are not optimal for Chilean student population. Factor analysis shows a structure of four-factor model being the best fit, but with problems in the fourth factor. Therefore, we suggest that the studied version of the scale needs improvement to ensure optimal indices of validation for Chilean population.

## Author Contributions

MG-A led the project and made part of the Introduction, Method, and Discussion. AM-M made part of the Introduction and the Discussion and led Method and Results. SG made part of the Introduction, part of the Method, and part of the Discussion. AU made part of the Introduction and the Discussion and led part of the Method.

## Conflict of Interest Statement

The authors declare that the research was conducted in the absence of any commercial or financial relationships that could be construed as a potential conflict of interest.
